# Lack of association between *CYB5A* gene rs1790834 polymorphism and the response to leflunomide in women with rheumatoid arthritis

**DOI:** 10.1007/s00228-021-03172-3

**Published:** 2021-06-23

**Authors:** Małgorzata Łączna, Damian Malinowski, Agnieszka Paradowska-Gorycka, Krzysztof Safranow, Violetta Dziedziejko, Andrzej Pawlik

**Affiliations:** 1 Department of Physiology, Pomeranian Medical University, 70-111 Szczecin, Poland; 2 Department of Experimental and Clinical Pharmacology, Pomeranian Medical University, 70-111 Szczecin, Poland; 3 Department of Molecular Biology, National Institute of Geriatrics, Rheumatology and Rehabilitation, 02-637 Warsaw, Poland; 4 Department of Biochemistry and Medical Chemistry, Pomeranian Medical University, 70-111 Szczecin, Poland

**Keywords:** Leflunomide, Therapy, Rheumatoid arthritis, Polymorphism, CYB5A

## Abstract

**Aim:**

Leflunomide is a disease-modifying antirheumatic drug used in therapy for rheumatoid arthritis (RA). Previous studies indicated that oestrogens and androgens may affect the response to leflunomide in RA patients. The synthesis of androgens is regulated by cytochrome CYB5A. The aim of this study was to examine the association between the *CYB5A* gene rs1790834 polymorphism and the response to leflunomide in women with RA.

**Methods:**

The study included 111 women diagnosed with RA. Leflunomide was administered in monotherapy at a dose of 20 mg/day. All patients underwent a monthly evaluation for 12 months after the initiation of treatment with leflunomide.

**Results:**

After 12 months of therapy, the changes in individual disease activity parameters, such as: DAS28, ESR, CRP and VAS, were not statistically significantly different between rs1790834 genotypes in the Kruskal–Wallis test.

**Conclusions:**

The results of our study suggest lack of statistically significant association between the *CYB5A* gene rs1790834 polymorphism and the response to leflunomide in women with RA.

## Introduction

Leflunomide is a disease-modifying antirheumatic drug used in therapy for rheumatoid arthritis (RA). Leflunomide is administered as a prodrug and is metabolised to malononitrilamide (MNA or A77 1726), a substance that exhibits biological activity [1]. Leflunomide works primarily by blocking dihydroorotate dehydrogenase (DHODH), an enzyme responsible for pyrimidine nucleotide synthesis [2]. It has been shown that DHODH gene polymorphisms may affect the efficacy of therapy with leflunomide [3, 4]. Leflunomide acts by inhibiting the proliferation of T cells and the activation of synovial macrophages. As a result, leflunomide has antiproliferative, anti-inflammatory, and immunomodulating properties [5–7]. The anti-inflammatory effects of leflunomide are related to the inhibition of the synthesis of proinflammatory cytokines by synovial cells and macrophages and the intensification of apoptosis of cells responsible for the development of inflammation in the joints [8–10]. The wide spectrum of action of this drug and the quite rare side effects make leflunomide a beneficial option for RA treatment.

RA is a disease that occurs more frequently in women, and worse treatment outcomes have been observed in women [11, 12]. The reason for this may be sex hormones (oestrogens and androgens), which can influence the activity and course of the inflammatory process in the joints of patients with RA [12, 13]. Previous studies have indicated that oestrogens and androgens may affect the response to leflunomide in RA patients [14–16]. Moreover, androgens exert anti-inflammatory properties [17, 18]. The synthesis of androgens is regulated by two enzymes of cytochrome P450c17: 17-alpha-hydroxylase and 17,20-lyase. The activity of 17,20-lyase is regulated by cytochrome CYB5A encoded by the *CYB5A* gene on chromosome 18 [19]. Previous studies have shown that the *CYB5A* gene rs1790834 polymorphism may alter the activity of cytochrome CYB5A [20]. This study aimed to examine the association between the *CYB5A* gene rs1790834 polymorphism and the response to leflunomide in women with RA.

## Patients

This study included 111 women with a mean age of 53.7 ± 10.2 years diagnosed with RA (duration of the disease, 11.3 ± 7.5 years). Leflunomide was administered in monotherapy at a dose of 20 mg/day. All patients underwent a monthly evaluation for 12 months after the initiation of treatment with leflunomide. The assessed variables included serum C-reactive protein (CRP), the erythrocyte sedimentation rate (ESR), the number of swollen and tender joints, the patient’s assessment of pain on a 100-mm visual analogue scale (VAS), and the disease activity score (DAS28). A 28-joint count (including the metacarpophalangeal joints, proximal interphalangeal joints, wrists, and elbows) was used. The study was approved by the ethics committee at Pomeranian Medical University, Szczecin, Poland, and written informed consent was obtained from all subjects.

## Genotyping

DNA was extracted from blood samples using the GenElute Mammalian Genomic DNA Miniprep Kit (Sigma-Aldrich, USA) according to the manufacturer’s protocol. All samples were genotyped in duplicate using an allelic discrimination assay on a CFX Connect Real-Time PCR Detection System (Bio-Rad, Germany) with TaqMan® probes.

## Statistical analysis

Distributions of the disease activity parameters were significantly different from normal (*p* < 0.05, Shapiro–Wilk’s test) in most cases; therefore, we used non-parametric tests. The Kruskal–Wallis test followed by the Mann–Whitney *U* test was used to compare disease activity parameters and their changes between genotype groups. A Wilcoxon signed-rank test was used for paired comparisons of parameters measured before and after the treatment; *p* < 0.05 was considered to be statistically significant.

## Results

The clinical parameters of patients are shown in Table 1. The distribution of *CYB5A* rs1790834 genotypes in the studied group of patients was as follows: GG 76 (68.47%), GA 29 (26.13%), and AA 6 (5.40%). These were in Hardy–Weinberg equilibrium (HWE) (*p* = 0.66).

**Table 1:** The baseline characteristics of patients

Parameters	*CYB5A* rs1790834								
	GG		GA		AA		*p* value		
	Median (Q1–Q3)		Median (Q1–Q3)		Median (Q1–Q3)		*p*^a^	GG vs GA + AA^b^	AA vs GG + GA^b^
Age (years)	54.0 (48.0–60.5)		50.0 (46.0–64.0)		53.0 (48.0–57.0)		0.85	0.57	0.88
Age of onset (years)	45.0 (34.0–51.0)		43.0 (38.0–49.0)		47.5 (38.0–48.0)		0.97	0.86	0.95
Duration of the disease (years)	9.5 (6.0–14.0)		8.0 (5.0–15.0)		8.0 (6.0–10.0)		0.71	0.41	0.80
	%		%		%		*p*^c^	GG vs GA + AA^c^	AA vs GG + GA^c^
RF positive	90.4		82.8		100.0		0.42	0.40	0.47
Anti-CCP positive	94.0		88.9		100.0		0.65	0.63	0.57
Erosive RA	94.7		96.6		83.3		0.43	0.93	0.21

*Anti-CCP* anti-cyclic citrullinated peptide antibody, *Q1* lower quartile, *Q3* upper quartile, *RF* rheumatoid factor

^a^Kruskal-Wallis test

^b^Mann-Whitney test

^c^Chi^2^ test

Table 2 presents the disease activity parameters before treatment with leflunomide. As shown, there were no statistically significant associations between the studied parameters and *CYB5A* rs1790834 genotypes. We only observed a tendency to lower DAS28 scores in RA patients with the A allele (*p* = 0.06). In Table 3, we show the disease activity parameters after 12 months of treatment with leflunomide. After 12 months of therapy, there were no statistically significant differences in disease activity parameters, such as DAS28, ESR, CRP and VAS in the Kruskal–Wallis test. Only in patients with GA and AA genotypes, DAS28 values were significantly lower than those with the GG genotype in the Mann–Whitney *U* test (*p* = 0.04). Table 4 shows the improvement in disease activity parameters during the 12 months of treatment with leflunomide in association with *CYB5A* rs1790834 genotypes. After 12 months of therapy, the changes in individual disease activity parameters, such as DAS28, ESR, CRP and VAS, were not statistically significantly different between rs1790834 genotypes in the Kruskal–Wallis test.

**Table 2:** The disease activity parameters before treatment with leflunomide in association with *CYB5A* rs1790834 genotypes

Parameters	*CYB5A* rs1790834					
	GG	GA	AA	*p* value		
	Median (Q1–Q3)	Median (Q1–Q3)	Median (Q1–Q3)	*p*^a^	GG vs GA + AA^b^	AA vs GG + GA^b^
ESR (mm/h)	50.0 (31.0–71.0)	42.0 (28.0–67.0)	56.5 (40.0–70.0)	0.28	0.36	0.39
CRP (mg/l)	30 (12.5–57.7)	26.7 (16.5–63.8)	37.4 (12.4–65.0)	0.86	0.60	0.76
VAS	8.0 (6.1–9.0)	8.0 (6.2–9.0)	7.5 (6.0–8.0)	0.70	0.63	0.43
DAS28	5.4 (5.0–5.8)	5.3 (4.7–5.5)	5.2 (4.8–5.7)	0.14	0.06	0.92

*Q1* lower quartile; *Q3* upper quartile

^a^Kruskal-Wallis test

^b^Mann-Whitney test

**Table 3:** The disease activity parameters after 12-month treatment with leflunomide in association with *CYB5A* rs1790834 genotypes

Parameters	*CYB5A* rs1790834					
	GG	GA	AA	*p* value		
	Median (Q1–Q3)	Median (Q1–Q3)	Median (Q1–Q3)	*p*^a^	GG vs GA + AA^b^	AA vs GG + GA^b^
ESR (mm/h)	29.5 (19.0–48.0)	30.0 (16.0–36.0)	25.0 (14.0–35.0)	0.47	0.25	0.45
CRP (mg/l)	6.0 (4.0–17.7)	5.0 (2.9–5.8)	5.0 (3.8–5.2)	0.26	0.10	0.64
VAS	2.0 (1.0–3.2)	2.0 (1.0–3.0)	1.0 (1.0–1.5)	0.34	0.36	0.19
DAS28	3.5 (2.9–4.1)	2.9 (2.5–3.8)	3.1 (2.7–3.8)	0.14	0.04	0.65

*Q1* lower quartile; *Q3* upper quartile

^a^Kruskal-Wallis test

^b^Mann-Whitney test

**Table 4:** The improvement of disease activity parameters after 12 months of treatment with leflunomide in association with *CYB5A* rs1790834 genotypes

Parameters	*CYB5A* rs1790834					
	GG	GA	AA	*p* value		
	Median (Q1–Q3)	Median (Q1–Q3)	Median (Q1–Q3)	*p*^a^	GG vs GA + AA^b^	AA vs GG + GA^b^
ESR (mm/h)	−15.0 (−34.0 to −2.0)^^^	−14.0 (−28.0 to −1.0)^	−37.0 (−100.0 to −18.0)	0.29	0.65	0.12
CRP (mg/l)	−13.4 (−40.5 to −3.7)^^^	−33.7 (− 66.0 to − 11.2)^^	−8.6 (−115.8 to −7.9)	0.16	0.06	0.80
VAS	−6.0 (−7.0 to −3.7)^^^	−6.0 (−6.8 to −5.0)^^	−7.0 (−7.5 to −4.0)	0.50	0.32	0.39
DAS28	−1.9 (−2.7 to −1.4)^^^	−1.9 (−2.7 to −1.5)^^	−2.6 (−2.7 to −2.0)	0.44	0.27	0.36

*Q1* lower quartile, *Q3* upper quartile

^*p* < 0.01; ^^*p* < 0.001; ^^^*p* < 0.00001, Wilcoxon signed-rank test for significance of change between the values before treatment and after 12 months of treatment; the test was not calculated for patients with AA genotype due to their low number

^a^Kruskal-Wallis test

^b^Mann-Whitney test

## Discussion

In this study, we examined the association between the *CYB5A* gene rs1790834 polymorphism and the response to leflunomide in women with RA. As previously shown, this polymorphism may change the expression of cytochrome CYB5A, which regulates androgen synthesis [20].

We analysed the disease activity parameters after 12 months of therapy. Comparing DAS28, ESR, CRP and VAS values between *CYB5A* genotypes, it was found that none of the *p* values obtained with the Kruskal–Wallis test was statistically significant, while only one comparison (DAS28) showed a marginally significant *p* value of 0.04 in the Mann–Whitney *U* test. It should also be noted that patients with the GG genotype had higher disease activity parameters before starting leflunomide treatment compared to patients with the GA and AA genotypes, including DAS28 (*p* = 0.06). DAS28, which includes the number of swollen and tender joints, and ESR is a EULAR response criterion commonly used in many clinical trials [21]. The ultimate goal of treatment for RA patients may be to achieve low disease activity (DAS28 less than 3.2) or reach a state of disease remission (DAS28 less than 2.6). The state of disease remission may be temporary and may require ongoing therapy. Therefore, it may be preferable to express a patient’s disease status as cumulative or average disease activity over some time, rather than classifying the patient as being in remission [22]. Our results indicate that the effect of the *CYB5A* rs1790834 polymorphism on the response to leflunomide treatment of RA patients is not significant. We observed it only in relation to DAS28. Previous studies have shown that other genetic polymorphisms, such as in the DHODH gene, oestrogen receptor gene and CYP1A2 gene, may be associated with the response to leflunomide in RA patients [4, 23, 24]. The response to treatment in patients with RA is determined by many factors, including genetics. Genetic polymorphism may be one of many factors influencing the results of the therapy. The effect of a single polymorphism on treatment outcomes seems to be limited and must be considered together with other polymorphisms and other factors affecting disease activity and response to treatment.

Previous studies have shown that the *CYB5A* rs1790834 gene polymorphism is associated with the risk of RA in women [20]. Women with the A allele had a lower risk of RA development. It was also demonstrated that the A allele caused an increase in CYB5A mRNA expression and increased activity of 17,20-lyase, the enzyme responsible for androgen synthesis. The activity of 17,20-lyase is regulated by CYB5A and the *CYB5A* gene polymorphism. Moreover, as was shown by Stark et al., higher levels of CYB5A may increase the synthesis of androgens [20]. These authors found that the A allele in RA patients may be responsible for increased density of cytochrome b5-positive cells in synovial tissue [20]. They demonstrated a two- to threefold increase in androgen synthesis in synovial fibroblasts from subjects with the *CYB5A* rs1790834 A allele, which corresponds to an increase in CYB5A expression [20]. Cytochrome b5 has a variety of functions that have different meanings in different tissues and cell types. The metabolism in the liver of fatty acids and the metabolism of steroids and xenobiotics depend on cytochrome b5 [25]. In another study, Stark et al. examined the effect of the *CYB5A* gene rs1790834 polymorphism on local endocrine function in joints. The authors indicated an association between the rs1790834 A allele and increased CYB5A expression in synovial fibroblasts responsible for androgen synthesis in the joints [26]. Xiang et al. investigated the association between *CYB5A* gene rs1790834 polymorphism and the efficacy of abiraterone acetate treatment in patients with castration-resistant prostate cancer. This therapy was more effective in patients with the *CYB5A* rs1790834 A allele [27].

Previous studies suggested that the response to leflunomide in women with RA is determined by sex hormones (oestrogens and androgens). Cutolo et al. investigated the effects of leflunomide, testosterone and 17β-estradiol on the synthesis of proinflammatory cytokines (IL-6, TNFα and TGF-β) in cultures of macrophages [14]. Leflunomide significantly inhibited the synthesis of cytokines. 17β-Estradiol significantly increased cytokine synthesis, whereas testosterone significantly decreased the production of cytokines. The authors observed that testosterone enhanced the inhibitory effect of leflunomide on cytokine synthesis in cultures of macrophages. The inhibition of cytokine production was significantly greater in cultures with leflunomide and testosterone than in cultures with leflunomide alone. Conversely, in cultures with 17β-estradiol and leflunomide, the authors observed less of a decrease in cytokine production than in cultures with leflunomide alone. The above results suggest that androgens may intensify the anti-inflammatory effects of leflunomide associated with the inhibition of proinflammatory cytokine synthesis, while oestrogens have the opposite effect [15]. Montagna et al. [16] investigated the effects of sex hormones (17β-estradiol and testosterone) on the pro-apoptotic properties of leflunomide on human macrophages. Cultures of macrophages were treated with leflunomide alone or with the addition of 17β-estradiol or testosterone. The authors indicated that leflunomide significantly enhanced the expression of apoptotic proteins. Testosterone significantly intensified these properties, while 17β-estradiol attenuated these properties [16]. The results indicate that androgens may potentiate the pro-apoptotic effect of leflunomide on inflammatory cells in the joints of RA patients.

RA is a much more common disease in women, possibly due to the effects of oestrogens. Studies have shown that oestrogens have an inflammatory effect in the synovial tissue of joints, while androgens have shown anti-inflammatory effects [12, 13, 17, 18]. Serum androgen concentrations are inversely correlated with disease activity parameters and disease severity. In the joint tissues of patients with the active form of RA, decreased androgen levels were found compared to patients with the inactive form of the disease [18]. In addition, increased aromatisation of androgens to oestrogens has been shown in cultures of synovial cells from RA patients [28].

The results of our study suggest lack of statistically significant association between the *CYB5A* gene rs1790834 polymorphism and the response to leflunomide in women with RA. Our study is limited by the number of patients; therefore, to confirm the influence of *CYB5A* gene rs1790834 polymorphism on the response to leflunomide in RA patients, numerous studies on a larger cohort of subjects should be performed.
